# Managing Postural Hypotension in Diabetic Autonomic Dysfunction When Adrenergic Drugs are Contraindicated: Case Report and Review of Literature

**DOI:** 10.7759/cureus.2039

**Published:** 2018-01-08

**Authors:** Syed Rizwan A Bokhari, Faisal Akhtar, Qurrat-Ul-Ain Abid, Uzma Jahanzaib, Maria R Bokhari, Sana Hasan, Khurshid Khan

**Affiliations:** 1 Department of Nephrology and Hypertension, Tulane University School of Medicine, New Orleans, LA; 2 Neurology, Ochsner Health System; 3 Internal Medicine, Jinnah Hospital Lahore (jhl) Pakistan; 4 Department of Internal Medicine, Division of Endocrinology, Mayo Hospital Lahore; 5 Department of Radiology, Tulane University School of Medicine, New Orleans, LA; 6 Department of Nephrology and Renal Transplant, Prince Sultan Medical Military City, Riyadh; 7 Department of Medicine, Jinnah Hospital, Allama Iqbal Medical College, Lahore

**Keywords:** diabetic neuropathy, nephrology, cardiology, internal medicine, autonomic neuropathy, dm, blood pressure, beta blockers

## Abstract

Postural hypotension, as a manifestation of autonomic neuropathy is a very sinister long-term debilitating complication of diabetes, is usually irreversible and tough to manage with medications. The treatment of this condition following the standard treatment protocols can be contraindicated in the patients with underlying heart conditions. We report the case of a patient at our hospital who presented with full-blown symptomatic dysautonomia secondary to long-standing diabetes, with bedside testing positive for autonomic dysfunction. Treating this patient with the standard protocol of adrenergic agonist could have worsened his underlying coronary artery disease. So, we moved a step aside to go out of the box and we have a trial of the β1-selective beta-blocker, with astonishing results and significant improvement in the quality of life and symptoms of postural hypotension. We report here the use of alternative treatment option in managing a patient with severe postural hypotension secondary to diabetes-related autonomic neuropathy when adrenergic drugs are contraindicated.

## Introduction

Orthostatic hypotension is the core feature of autonomic failure. Orthostatic hypotension is defined as a fall in the blood pressure of 20/10 mmHg on standing for three minutes. This definition is meant to capture the initial or mild cases. The symptomatic orthostatic hypotension is more commonly encountered in the subjects with falls in blood pressure that may range from this figure to the severe cases where the fall in the blood pressure may be 100/50 mmHg. The most common symptoms of orthostatic hypotension are lightheadedness, mining of vision, and discomfort in the head (typically occipital), neck, shoulders, and sometimes the chest. Orthostatic hypotension is the most bothering symptom, often complained by the patients suffering from diabetic neuropathy [1]. Many patients with orthostatic hypotension can be treated by increasing the plasma volume and by increasing the fluid and sodium intake to improve the orthostatic tolerance or can be treated pharmacologically by giving fludrocortisone with sympathomimetic agents [2].

Diabetic neuropathy can be asymptomatic or can be a cause of significant disability. Any or all the organs innervated by the autonomic system can be affected by diabetes. The symptoms can arise from the involvement of a single system or multiple systems and can range from postural hypotension, diarrhea, gastroparesis, overflow urinary incontinence, impotence to dry skin and gustatory sweating [3]. Commonly instituted tests used to detect the presence of dysautonomia are:

1) Beat-to-beat cardiac variation with position or respiration

2) Valsalva ratio (normal-longest to shortest relative risk (R-R) ratio > 1.2)

3) Heart rate response to deep breathing (normal-E: I ratio ≥ 1.17)

4) Ratio being 30:15 R-R Interval ratio (30th to 15th R-R ratio on standing > 1.03)

5) Fall in the blood pressure of 20/10 mmHg on standing for three minutes.

## Case presentation

Diabetic autonomic neuropathy often goes unrecognized and it remains asymptomatic most of the time, but often it presents with debilitating symptoms. We present a case of 48-year-old male with the history of a type-II diabetic for 20 years and coronary artery disease (CAD). The patient had poor control of diabetes and had diabetic complications of gastroparesis, retinopathy, nephropathy, neuropathy, and CAD. The presenting complaints were dizziness for two months with the progressive inability to stand without support. On examination, he was a lethargic middle-aged man lying comfortably in bed. The lying blood pressure (BP) was 150/90 mmHg with the significant postural drop measuring up to 80/60 mmHg after two minutes of standing. The neurological exam of diabetes showed distal symmetric sensory neuropathy to light touch, while the vibration sensation and proprioception were intact. The fundoscopy revealed scattered hard exudates, dot, and blot hemorrhages. He was never tested for autonomic dysfunction prior to this encounter and did not take any medication, since midodrine was not available in Pakistan and fludrocortisone was never prescribed by the general practitioner, due to the lack of complete testing and affordability issues.

Electrocardiography findings suggestive of dysautonomia were:

1) Abnormal heart rate response to deep breathing was 1.0 (normal-E: I ratio ≥ 1.17)

2) The abnormal Valsalva ratio was 1.03 (normal-longest to shortest R-R ratio > 1.2)

3) Abnormal 30:15 R-R interval ratio was 0.99 (30th to 15th, R-R interval ratio on standing > 1.03).

These three tests along with the significant postural drop were suggestive of marked cardiac dysautonomia in this patient. The lab data showed the complete blood count, serum electrolyte test, renal function tests, liver function tests, serum cortisol, Adrenocorticotropic hormone (ACTH) and the thyroid function tests as normal. Midodrine, in this patient, could not be used because of the history of the significant CAD. The patient did not have any response to fludrocortisone. So as a final resort, he was started on atenolol P.O. (initial dose of atenolol 12.5 mg b.i.d. which was gradually increased to 25 mg b.i.d.). The patient had dramatic clinical improvement with this therapy. The orthostatic blood pressure changes improved and he was able to ambulate without any difficulty.

## Discussion

Diabetic autonomic neuropathy is the impairment of autonomic control in the setting of diabetes after exclusion of other causes. Ewing, et al. recommend five simple tests, the cardiac autonomic reflex tests to establish the diagnosis: 1) Heart rate variability (HRV) with deep breathing; 2) the HRV lying to standing; 3) the Valsalva maneuver; 4) postural fall in the blood pressure; and 5) the blood pressure response to sustained handgrip [4]. A single abnormal test may indicate early cardiac autonomic neuropathy and three positive tests are recommended for a definite diagnosis. The diabetic autonomic neuropathy can be quite challenging and difficult to treat. Although medications cannot cure autonomic neuropathy, they can help with finding the symptoms.

Drugs used for the treatment of postural hypotension

Fludrocortisone

Fludrocortisone is a mineralocorticoid, which causes sodium retention and enhances the sensitivity of the blood vessels to circulating catecholamine [5].

Midodrine

Midodrine is a prodrug that acts by stimulating the alpha-1 adrenoreceptors to constrict the blood vessels and raise the blood pressure [6]. The pressor effect of midodrine comes on within about 30 minutes and wanes after four or five hours.

Even though adrenergic drugs like midodrine are the first line choice in the patients with orthostatic hypotension from diabetic autonomic neuropathy, their use is limited in the patients with contraindications.

The standard protocol of treating diabetic autonomic neuropathy related postural hypotension recommends using fludrocortisones and midodrine, however, our patient was having coronary artery disease, so the use of adrenergic agonist was just synonymous to relieving one debilitating symptom and putting him into another life-threatening state. The studies have shown that the beta-adrenergic blockers do not cause orthostatic hypotension [7]. A cardio-selective β1-blocker, atenolol 12.5 mg was started twice daily, which was gradually increased to 25 mg b.i.d. After some time, the patient reported significant improvement in his postural hypotension.

Nonselective β-blockers with intrinsic sympathomimetic activity and the β1 selective agents with no intrinsic sympathomimetic activity like atenolol have been used in several trials in the treatment of orthostatic hypotension and for the prevention of presyncope with variable results [8]. The suggested mechanism of action of these agents is the blockade of the β2 receptors, allowing unopposed adrenoreceptor mediated vasoconstriction [7]. Moreover, the beta-blockers can help regulate the heart rate and rhythm with beneficial effects for the patients with coronary artery disease and ischemic heart disease [9]. However, we noticed a remarkable role of the beta-blockers in the treatment of postural hypotension due to diabetic autonomic dysfunction.

A similar case was reported in the Journal of Human Hypertension in 2006. A 54-year-old male with diabetes was referred to the hypertension clinic in the Jichi Medical School Hospital with symptoms of dizziness and orthostatic intolerance and was successfully treated with the β1-blocker [10].

The β1- selective blocker (atenolol) effectively reduced the orthostatic blood pressure changes in this patient with severe orthostatic hypotension due to diabetic autonomic neuropathy and the main finding was an improvement of the elevated supine blood pressure without reducing the standing blood pressure. According to the author, this differential antihypertensive effect is ideal for the medication in orthostatic hypotension, but the authors found it to be very different from other case reports, of the effects of the β-blockers, where the emphasis has been on increasing the standing pressure. They claim that the study was unique in two ways: 1) the use of 24-hr ambulatory monitoring and 2) they describe a potentially different mechanism whereby the β-blockers may benefit the patients with orthostatic hypotension.

## Conclusions

Postural hypotension, as part of the spectrum of diabetic autonomic dysfunction, can be difficult to manage in conditions where adrenergic drugs are contraindicated. Our case report highlighted the importance of the beta blockers for the treatment of postural hypotension in special circumstances. Increased awareness and effective monitoring of diabetic autonomic neuropathy can help decrease the morbidity associated with it. Further studies are needed to address this cumbersome condition.
